# Short-Term Magnesium Therapy Alleviates Moderate Stress in Patients with Fibromyalgia: A Randomized Double-Blind Clinical Trial

**DOI:** 10.3390/nu14102088

**Published:** 2022-05-17

**Authors:** Nicolas Macian, Christian Dualé, Marion Voute, Vincent Leray, Marion Courrent, Paula Bodé, Fatiha Giron, Sylvie Sonneville, Lise Bernard, Fabienne Joanny, Katell Menard, Gilles Ducheix, Bruno Pereira, Gisèle Pickering

**Affiliations:** 1 Platform of Clinical Investigation Department, INSERM CIC 1405, University Hospital Clermont-Ferrand, F-63000 Clermont-Ferrand, France; cduale@chu-clermontferrand.fr (C.D.); mgauffier@chu-clermontferrand.fr (M.V.); vleray@chu-clermontferrand.fr (V.L.); mcourrent@chu-clermontferrand.fr (M.C.); apbode@chu-clermontferrand.fr (P.B.); fraki@chu-clermontferrand.fr (F.G.); ssonneville@chu-clermontferrand.fr (S.S.); katell.menard@hotmail.fr (K.M.); gducheix@chu-clermontferrand.fr (G.D.); gisele.pickering@uca.fr (G.P.); 2 INSERM 1107, University Clermont Auvergne, F-63000 Clermont-Ferrand, France; 3 Clinical Research/Temporary Authorization Department, University Hospital Clermont-Ferrand, F-63000 Clermont-Ferrand, France; l_bernard@chu-clermontferrand.fr; 4 FJ Recherche et Developpement, Research Organization, 230 Rue du Faubourg Saint-Honoré, F-75008 Paris, France; fabienne.joanny@fj-lifesciences.com; 5 Clinical Research and Innovation Department, University Hospital Clermont-Ferrand, F-63000 Clermont-Ferrand, France; bpereira@chu-clermontferrand.fr

**Keywords:** stress, magnesium, fibromyalgia, pain, magnesium supplementation

## Abstract

Patients suffering from fibromyalgia often report stress and pain, with both often refractory to usual drug treatment. Magnesium supplementation seems to improve fibromyalgia symptoms, but the level of evidence is still poor. This study is a randomized, controlled, double-blind trial in fibromyalgia patients that compared once a day oral magnesium 100 mg (Chronomag^®^, magnesium chloride technology formula) to placebo, for 1 month. The primary endpoint was the level of stress on the DASS-42 scale, and secondary endpoints were pain, sleep, quality of life, fatigue, catastrophism, social vulnerability, and magnesium blood concentrations. After 1 month of treatment, the DASS-42 score decreased in the magnesium and placebo groups but not significantly (21.8 ± 9.6 vs. 21.6 ± 10.8, respectively, *p* = 0.930). Magnesium supplementation significantly reduced the mild/moderate stress subgroup (DASS-42 stress score: 22.1 ± 2.8 to 12.3 ± 7.0 in magnesium vs. 21.9 ± 11.9 to 22.9 ± 11.9 in placebo, *p* = 0.003). Pain severity diminished significantly (*p* = 0.029) with magnesium while the other parameters were not significantly different between both groups. These findings show, for the first time, that magnesium improves mild/moderate stress and reduces the pain experience in fibromyalgia patients. This suggests that daily magnesium could be a useful treatment to improve the burden of disease of fibromyalgia patients and calls for a larger clinical trial.

## 1. Introduction

Fibromyalgia (FM) affects 2% of the general population, with a predominance in women [1]. FM is characterized by widespread chronic pain and patients report symptoms, including fatigue, muscle pain, sleep disorders, anxiety, depression, cognitive dysfunction [2,3], and stress [4,5].

Stress has been defined as a state of acute or chronic clinical disturbance of the body’s homeostasis related to various stressors, which could have a psychological (anger, anxiety, depression) or biological (infection, burn, etc.) origin [6,7,8]. In response to stressors, a set of adaptive physiological processes are initiated that aim to restore homeostasis. The physiological mechanisms of the stress response involve the hypothalamic-pituitary-adrenal (HPA) axis and the autonomic nervous system (ANS). These mechanisms interact with each other and have positive feedback loops at different levels [6,9]. If the capacity of the stress response system to adapt is overwhelmed, chronic diseases may then appear [7].

FM has also been described as a stress-related trouble with abnormal functioning of the HPA axis [9,10,11] and with a hyporeactivity to physical and mental stressors [10,11]. Studies have reported that 76.7% of FM patients experience stress [12] and have more comorbidities than matched controls [4,13]. Strong correlations between stress and pain in patients suffering from FM have been described [14].

Medication (antidepressants, anxiolytics) is prescribed for stress, but this has significant adverse events on the central nervous system that may impact the patient’s quality of life [15,16]. Non-drug approaches are therefore recommended and among nutrients, magnesium (Mg) is known to be involved in the vicious circle of stress [8,17,18]. Mg participates in many metabolic reactions, energy production, synthesis of nucleic acid and protein [19], neuronal transmission, neuromuscular function, and regulation of the cardiac rhythm, and plays a key role in membrane excitability [8]. Mg acts also on the N-methyl-D-aspartate (NMDA) receptor, which plays a pivotal role in pain and mental functioning [20]. Hypomagnesemia may induce a decrease in melatonin, thus causing sleep disturbances, but also induces an increase in substance P, increasing pain and stress-related hormones [11,21]. A lack of Mg is suspected in FM patients, who may have low levels of Mg in plasma and hair [22,23,24,25,26,27] and in their diet [28].

Mg supplementation has been suggested to relieve the various symptoms associated with FM [22,23,25,29,30,31,32], reducing certain types of pain and improving the ability of the central nervous system to withstand stress [22,23,24,30,33]. Recent studies have reported that up to 36% of FM patients take Mg supplementation [15,34], but so far, no studies have explored the impact of oral Mg on stress in patients suffering from FM.

Considering the paucity of data in the literature and the need to identify therapeutic options for FM patients who often face treatment failure, our hypothesis is that Mg supplementation could improve stress and quality of life in this population. This randomized clinical trial (RCT) aimed to evaluate the impact of Mg in patients with FM on (i) stress and (ii) other FM symptoms, including pain, sleep, quality of life, fatigue, catastrophism, social vulnerability, and Mg blood concentrations.

## 2. Materials and Methods

### 2.1. Study Design

This study was a prospective double-blind RCT in FM patients with 2 parallel groups (Mg vs. placebo). It was carried out in the Platform of Clinical Investigation Centre, University Hospital, Clermont-Ferrand, France. This study took place from April 2019 to May 2020. The study was organized into 4 visits: an inclusion visit, randomization and initiation of treatment (D0), an end-of-treatment visit (D0 + 28 days, (D28)), and a follow-up visit (D0 + 84 days, (D84)).

### 2.2. Study Population

All patients gave their informed consent before participation in the study. To be included, patients had to be over 18 years of age with a confirmed diagnosis of FM, and have a stress score of over 18 on the Depression Anxiety Stress Scale-42 (DASS-42). Exclusion criteria were women with childbearing potential who were not using an effective contraceptive, woman who were pregnant or breastfeeding, contraindication to Mg treatment, diabetes mellitus, plasma Mg > 1.05 mmol·L^–1^, renal failure (creatinine clearance < 30 mL·min^–1^), treatment with a dietary supplement containing Mg, stable on their current treatment for at least 3 months, treatment with antibiotics, inclusion in another interventional trial, and unable to understand the patient information and informed consent form.

### 2.3. FM Diagnosis, Impact of FM, and Pain Status

FM diagnosis was achieved using the ACR 2016 criteria [35] at the inclusion visit. The impact of FM was assessed using the Fibromyalgia Impact Questionnaire (FIQ; average FM > 50, severe FM > 70) [36] and the Fibromyalgia Rapid Screening Tool (FIRST; a patient suffering from FM has a FIRST score ≥ 5) [37].

### 2.4. Study Treatment

Patients were randomized to receive a 28-day treatment of either oral Mg (Chronomag^®^, Mg chloride technology formula) 2 tablets of 50 mg once a day (batch number EU1701), or an oral placebo (lactose).

Chronomag^®^ magnesium technology is an advanced patented low-dose formulation in a specific matrix specifically designed to provide a single daily intake of 100 mg of Mg element (vs. the current recommended dose of 300 mg/day) by providing a unique continuous low-dose Mg release. This continuous ‘low-dose’ Mg release throughout the gastrointestinal tract is in line with the natural physiological absorption process. Thus, this technology improves bioavailability and gastrointestinal tolerance.

Treatment allocation followed a predefined randomization plan and was conducted by a person independent of the protocol. The randomization sequence was generated using random blocks. Mg tablets and lactose tablets as the placebo (identical to Mg) were provided, purchased, and packaged (in an identical container) by the Central Pharmacy of the University Hospital of Clermont-Ferrand. To maintain good compliance and to follow adverse events (AEs), patients were contacted via a phone call once a week by a blinded clinical research assistant. Patients were included in this study on the condition that they accepted they would not start any new drug treatment (including magnesium supplements), consume any supplementary drug or water containing magnesium, and would not change their level of exercise during the whole study period. These confounding factors were controlled for during the study with the completion of a diary (from day 0 to day 84), where the patient was requested to record any deviation from the recommendations. After analysis, it was observed that all patients respected the recommendations.

### 2.5. Primary Outcome

The primary outcome was the stress score at D28, assessed by the DASS-42. This questionnaire is a self-report instrument designed to measure the three related negative emotional states of depression, anxiety, and stress, and we only considered the dimension of stress [38] for inclusion. The stress class is scored as follows: 0–14 (normal), 15–18 (mild, (m)), 19–25 (moderate (M)), 26–33 (severe (S)), and 34 and more (extremely severe (S+)). DASS-42 measures were carried out at every visit.

### 2.6. Secondary Outcomes

All measures of the secondary outcomes were carried out on D0, D28, and D84 visits.

Pain was assessed using a 11-point pain numerical rating scale (NRS) (ranging from 0 = no pain to 10 = the worst pain possible) and the Brief Pain Inventory (BPI) [39], a questionnaire comprising 9 items that assesses, as a recall of the 2 past weeks, (i) different aspects of pain intensity and (ii) the interference of pain on the physical and psychosocial aspects of daily life. The patient rates each question on a scale from 0 to 10.

Quality of sleep was assessed using the Pittsburgh Sleep Quality Index (PSQI) [40]. This questionnaire comprises 19 items that assess the following 7 domains: subjective sleep quality, sleep latency, sleep duration, habitual sleep efficiency, sleep disturbances, use of sleep medication, and daytime dysfunction. Each domain is scored from 0 to 3. The global score is a sum of the 7 domains and varies from 0 to 21, with a high score representing a greater alteration in the quality of sleep.

Quality of life was assessed using the 12-item Short Form Survey (SF-12) [41], which is a multipurpose short form survey with 12 questions, all selected from the SF-36 Health Survey [42]. The SF-12 is a generic measure and does not target a specific age or disease group. The SF-12 is weighted and summed to provide easily interpretable scales for physical and mental health. Then, an index of 0 to 100 is determined for mental and physical health. 

Fatigue was evaluated using the Fatigue Severity Scale (FSS), a 9-item self-questionnaire used to identify fatigue intensity. Each score varies from 1 to 7 for each question, with a low value representing a low intensity [43].

Catastrophism was assessed using the Pain Catastrophizing Scale (PCS) [44], a 13-item questionnaire allowing the patient to describe their thoughts and emotions during pain. The patient indicates to what extent she has these thoughts or emotions when she feels pain by assigning a score between 0 (not at all) and 4 (all the time) to each item. The total score is the sum of the scores for each question. A high value represents higher catastrophism.

Social vulnerability was assessed by a French questionnaire Evaluation de la Précarité et des Inégalités de Santé dans les Centres d’Examens de Santé (EPICES). This questionnaire is composed of 11 binary questions indicating precariousness and health inequalities, which are scored from 0 to 100. A patient is socially vulnerable if the EPICES score is ≥ 30.2 [45].

Mg assays were assessed by collecting a total of 30 mL of venous blood from each patient in a heparin tube with gel (for serum Mg) or heparin tube (for erythrocyte Mg). The tubes were sent to the biochemistry department of the University Hospital of Clermont-Ferrand for assay.

Serum Mg levels were analyzed using the Dimension Vista1 System Flex1 Mg reagent cartridge (Siemens Healthcare Diagnostics Inc, Deerfield, IL, USA). This is a modified version of the methylthymol blue complexometric procedure. Methylthymol blue forms a blue complex in the presence of Mg and the amount of complex formed can be measured using a bichromatic endpoint method to determine the concentration of Mg.

The erythrocyte Mg concentration was measured using a colorimetric method based on the formation of a colored complex of Mg with xylidyl blue reagent, in alkaline solution (Eurofins Biomnis, Lyon, France).

Mg measurement was also carried out at the inclusion visit.

### 2.7. Statistical Methods

All prespecified analyses were performed before the randomization code was broken. Continuous data were expressed as mean ± SD. The assumption of normality was assessed with the Shapiro–Wilk test. To evaluate the impact of Mg on stress, a mixed model for repeated data was constructed, with time, group, and their interaction as fixed effects and subject as a random effect, to model the between- and within-patient variability. A Sidak’s type I error was applied to take into account multiple comparisons between time points. Multivariable analyses were performed to take into account possible confounding variables (especially medical history and treatments) despite the randomization.

A post hoc subgroup analysis was conducted to test the difference in the Mg effect according to the stress level at inclusion (DASS stress score ≤ >25). We distinguished 2 subgroups: mild/moderate (m/M) with DASS-42 stress score < 25 and severe/extremely severe (S/S+) with DASS-42 stress score ≥ 25 according to the European Medical Agency (EMA) guidelines [46]. *p*-values of the interaction were derived from the multivariable random effect model including the randomized group (Mg or placebo) and an interaction term. The results were also expressed using effect sizes and 95% confidence intervals and represented using a forest plot.

For comparisons concerning non-repeated measures, the Student’s *t*-test and Mann–Whitney test (when assumptions required for the *t*-test were not met) were used for quantitative variables. Homoscedasticity was analyzed using the Fisher-Snedecor’s test. For categorical data, the chi-square and Fisher’s exact tests were performed. 

The statistical analyses were performed using Stata software version 15 (StataCorp, College Station, TX, USA). Statistical tests were 2-sided with the type-I error set at 5%. Because of the potential for type I error due to multiple comparisons, findings from the analyses of secondary endpoints should be interpreted as exploratory.

The sample size was estimated to highlight the absolute difference in stress from DASS equal to 3 points for a standard deviation (SD) of 4 [47], a 2-sided type I error at 5%, and 90% statistical power. To show such an impact of Mg on stress, it was necessary to include 38 patients in each group.

## 3. Results

### 3.1. Clinical Characteristics

**Figure 1:**
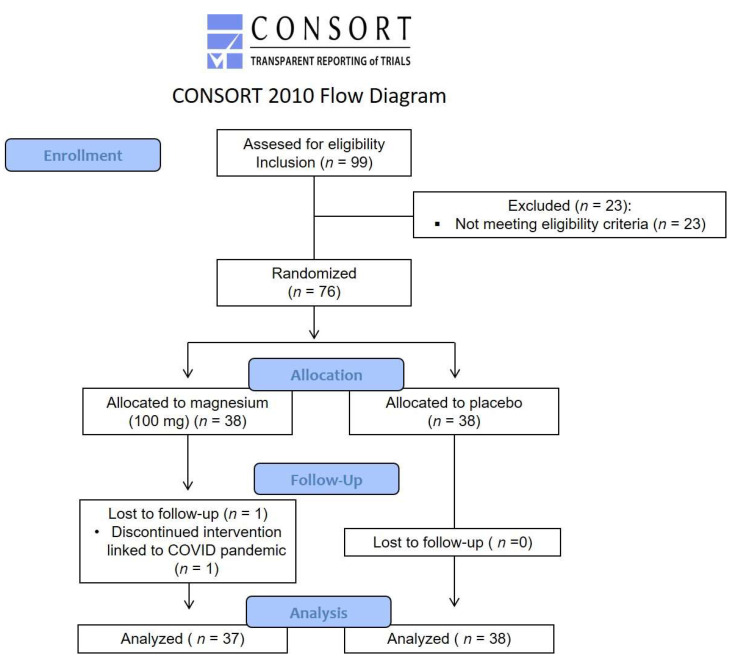
Flow study diagram.

From April 2019 to March 2020, patients with FM were screened, then included and randomized according to the flow study diagram (Figure 1).

Table 1 summarizes the baseline characteristics of the patients at inclusion. Medical history and concomitant medications at inclusion are presented in Table S1.

### 3.2. Primary Outcome Results

At inclusion, the DASS-42 score did not differ between the two groups for Mg vs. placebo. After 1 month of treatment, the DASS-42 score decreased in both groups but was not significantly different (21.8 ± 9.6 vs. 21.6 ± 10.8, respectively, *p* = 0.930).

**Figure 2:**
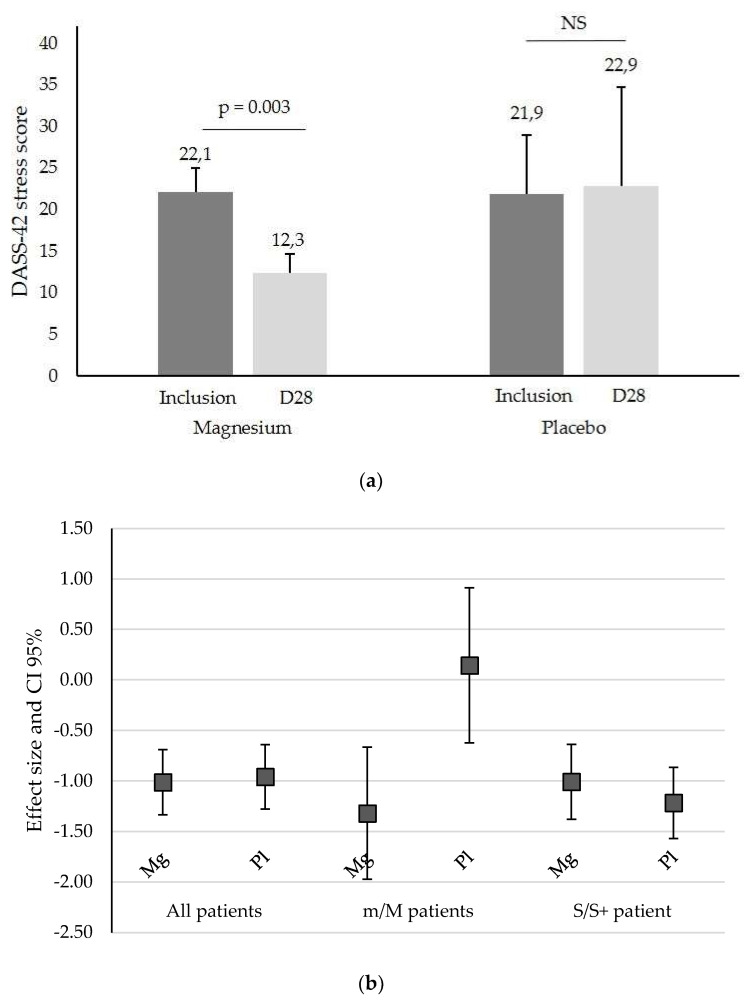
(**a**) DASS-42 stress score (mean ± SD) in patients with a mild/moderate (m/M) stress score at inclusion and after one month of Mg or placebo treatment (D28), NS, not significant. (**b**) DASS-42 stress effect size of Mg and placebo (Pl) for all patients, mild/moderate (m/M) patients, and severe/extremely severe (S/S+) stress at inclusion. The squares represent the effect size (variation in the DASS-42 value) and the whiskers represent its 95% CI limits.

However, when the population was separated between the m/M and S/S+ subgroups according to their stress at inclusion, the interaction term of baseline vs. intervention period differences was significantly different between randomization groups. Mg significantly reduced the stress score in the m/M group (22.1 ± 2.8 to 12.3 ± 7.0 for Mg vs. 21.9 ± 2.3 to 22.9 ± 11.9 for placebo, *p* = 0.003, Figure 2a) but not in the S/S+ group (33.6 ± 4.1 to 24.9 ± 8.3 for Mg vs. 32.3 ± 4.4 to 21.4 ± 10.7 for placebo, *p* = 0.320, Figure 2b).

### 3.3. Secondary Outcomes Results

**Figure 3:**
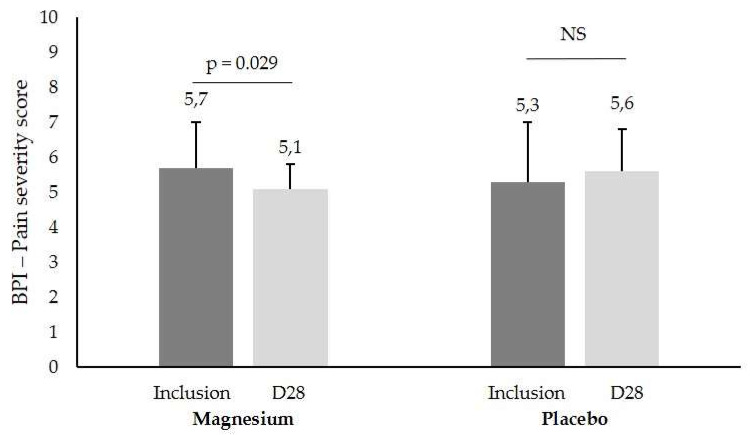
BPI ‘pain severity’ score (mean ± SD) in patients with a mild/moderate (m/M) stress score at inclusion and after one month of Mg or placebo treatment (D28), NS, not significant.

Pain evaluated as ‘pain severity’ in the BPI showed a significant difference between Mg and placebo (5.7 ± 1.3 to 5.1 ± 1.7 vs. 5.3 ± 0.7 to 5.6 ± 1.2, respectively, *p* = 0.029, Figure 3) in m/M patients. The FM duration, age of patient, and pain parameters according to stress at inclusion are summarized in Table 2.

All other results were not significantly different, including other pain measures, sleep quality, quality of life, fatigue, and catastrophizing precariousness. The results are presented in Table S1.

Plasma and erythrocyte Mg concentrations were not significantly different after one month of treatment; the results are summarized in Table 3.

No significant differences regarding the initiation of new treatment during the study between groups are reported (including antiepileptics, antidepressants, and anxiolytics). The results are summarized in Table 4.

Concerning drug tolerance, 30% of the patients in the Mg group and 31% of the patients in the placebo group reported minor gastrointestinal disorders (abdominal pain, diarrhea, etc.) during the study.

Finally, regarding compliance with the dietary recommendations, no deviations were reported.

## 4. Discussion

The main objective of this study was to explore whether one month of treatment with Mg could alleviate stress in patients suffering from FM. The results show that Mg does not change the stress severity in the overall sample vs. placebo but, for the first time, that Mg supplementation significantly reduces stress in mild to moderately stressed patients (*p* = 0.003) but not in severely stressed FM patients.

This finding is interesting as FM, a stress-related disorder, is described to result in exacerbation of the syndrome in stressful periods [9,18]. The efficacy of Mg on stress among moderately stressed patients may be explained by different factors: Mg specificities and dosage, a different state of dysregulation of homeostasis, and/or a different placebo effect in moderately or severely stressed persons.

The mechanism of action of Mg in general and on stress in FM is complex [32]. The Mg formula, Chronomag^®^, was chosen as it is a low dose of Mg chloride, with excellent absorption and bioavailability [48], which means that Mg is readily available to cross the cell membranes [48]. FM patients had baseline Mg serum and erythrocyte concentrations (0.92 ± 0.08/2.86 ± 0.32 mmol/L for Mg group vs. 0.91 ± 0.08/2.97 ± 0.31 mmol/L for placebo group) that were within the reference range (0.66–1.07/2.22–3.51 mmol/L), and they were not hypomagnesemic. After the intervention, Mg serum concentrations were 0.87 ± 0.07/2.87 ± 0.37 mmol/L for the Mg group vs. 0.87 ± 0.08/2.91 ± 0.37 mmol/L for the placebo group (*p* = 0.422/0.875). This finding is different from some publications, in which FM patients were reported to have hypomagnesemia and lower erythrocyte Mg concentrations than controls [23,27,49,50]. In practice, an Mg deficiency is difficult to identify because serum levels are often compensated by the release of Mg from the bone reservoir [51]. The literature has also reported nutritional deficits/disorders/adaptations in FM persons with changes in their dietary Mg intake [28,52]. In fact, people may have a chronic negative Mg balance without obvious measured hypomagnesemia [51,53], and may still benefit from Mg supplementation [54]. A recent study, with no placebo group, in 264 healthy adults with low magnesemia showed that oral Mg supplementation alleviated stress, and that severe stress was improved by Mg B6 in 176 persons [55]. Interestingly, the estimated average requirement of Mg was negatively correlated with DASS-42 stress scores [56]. In our study, the dosage of 100 mg per day may have been far too low to compensate an Mg deficit in severely stressed persons while it was adequate in moderately stressed FM persons.

FM patients are known to experience dysregulation of many systems, and they oscillate to maintain homeostasis when confronted with an overload [7]. FM may also impair cognition, as witnessed by clinical evaluation, and FM patients may underestimate their own scores, situation, and capacities. This dysregulation of homeostasis may be reduced in moderate compared to severe stress. In moderate stress, FM patients may be in a reversible stress situation and the impact of Mg at the cellular and molecular levels is active and efficient. On the contrary, with severe stress and a more robust allostatic situation, it is more difficult to switch to a lower level of stress. It is thus not surprising that in our trial, FM patients with severe pain showed no alleviation of stress, suggesting, on the one hand, a limited effect of Mg on severe stress and, on the other hand, the loss of homeostatic regulation capability at the central level [57,58,59].

Another intervening factor may be the different placebo effects on stress between the moderately and severely stressed groups. A placebo effect in FM/chronic pain has been described in the literature [60,61], with lower effects shown after long-term exposure to FM [62,63]. In our study, the duration of the disease cannot be incriminated as the FM duration was slightly longer in the S/S+ group than in the m/M group but not significantly different (9.3 ± 7.0 vs. 6.1 ± 5.1 years; *p* = 0.09), similarly to pain and pain severity. The age of FM patients may also be at play as a study showed that the placebo effect increased with age in FM patients [63]. In our study, 50% of the patients were over the age of 50 years in the m/M group whereas in the S/S+ group, 66% of the patients were over the age of 50 years. Neuroimaging studies have revealed the anatomical proximity of brain areas controlling stress, emotion, cognition [64,65,66,67], and pain [68,69,70,71], and a placebo effect has been visualized in fMRI, where the alleviation of pain and/or negative feelings have been implicated [72,73,74]. Pain itself involves cognitive-emotional aspects and interacts with stress [64,75], and stress is largely present in many chronic pain syndromes and may increase and maintain pain in FM [76]. Another study, exploring transcranial direct current stimulation (tDCS) and its impact on quality of life (QoL) using the SF-36 questionnaire, observed a non-specific effect on this parameter, which may be due to the placebo [77]. Regarding pain severity, a review reported that an increasing placebo effect was more likely when the patient presented with a higher baseline pain severity [63]. Our study also focused on pain parameters, a major complaint in FM. The effect of Mg on pain has been studied in recent reviews [78,79], showing a global modest effect of Mg; however, there is a lack of good-quality RCTs in the literature, as only three RCTs have been published on FM [22,23,29]. In our study, patients with mild/moderate stress reported a lesser pain severity after one month of treatment. This finding confirms the literature on FM, where decreases in the tender point index (Abraham and Flechas, 1992), the number of tender points (15.3 ± 2.5 to 11.7 ± 6.8, *p* = 0.032) [23], and the pain/tenderness measure [29] have been reported, although other studies did not confirm these results [80,81].

The pain diminution from 5.7 ± 1.3 to 5.1 ± 1.7 on the BPI pain severity scale observed in our study, although statistically significant, appears marginal compared to studies with pharmacological therapies, such as pregabalin [82,83,84], duloxetine [85,86], or amitriptyline [87]. These studies have reported, for example, the following impacts on the WPI [86] or pain scores: using the Visual Analogic Scale, an effect size of 0.38 at week 6; using the BPI pain severity scale, an effect size of 0.36 at week 6 [82] with pregabalin; and a difference of −0.43 with amitriptyline [87]. Furthermore, these drugs had adverse drug reactions [82], such as dizziness, lightheadedness, and dry mouth, with patient withdrawals, and the treatment duration was much longer. Moreover, in its recommendations [88], the European League Against Rheumatism stresses that first-line treatment of FM should involve exercise and patient education and should focus on non-pharmacological therapies, and not drugs. Considering these guidelines, we suggest that this alleviation of BPI pain severity, encompassing worst pain, least pain, average pain, and pain now, and triggering no adverse events and no patient withdrawals, is clinically pertinent for FM patients using a dietary supplement.

Concerning the other endpoints, including other pain measures, sleep quality, quality of life, fatigue, and catastrophizing precariousness, no significant differences were demonstrated between groups. Concerning sleep disturbances, which is a major symptom in FM, standardized recommendations (RDA) established by the National Academy of Sciences indicate that 375 mg of Mg per day is not adequate for improving sleep quality in women with FM [89]. A recent review is consistent with our results and indicates that RCTs showed an uncertain association between magnesium supplementation and sleep disorders [90]. Our negative results regarding the secondary outcomes are probably due to the sample size (power not based on these endpoints) and/or doses or duration of treatment, which are identified as study limitations.

## 5. Conclusions

This double-blind RCT indicates that although a non-significant reduction in stress was shown after one month of Mg supplementation, stress improved in moderately stressed FM patients and reduced the pain experience compared to the placebo. Mg is hence useful for a subgroup of FM patients with a moderate level of stress and pain. This is in line with previous studies of our group [91,92], with different responses to antidepressants, where we identified subgroups of FM responders. As mentioned previously, FM has been described as a stress-related trouble, with the HPA axis being implicated, and the impact of daily stress in this population could enhance the vicious circle of stress, increasing magnesium loss, causing a deficiency, and, in turn, enhancing susceptibility to stress [8]. We believe that the alleviation of stress and pain, in moderately impaired persons suffering from FM, achieved from using a non-drug treatment for one month may improve patients’ daily life. These findings represent a good basis for further clinical studies, with a possible demonstration of benefits not only on stress and pain but also fatigue and quality of life, including more patients and with a longer duration of treatment in FM and other complex clinical situations.

## Figures and Tables

**Table 1:** Baseline characteristics of patients with FM (*n* = 75).

	Mg (*n* = 37)	Placebo (*n* = 38)
Age (years, mean ± SD)	54.0 ± 11.5	51.8 ± 10.9
BMI (mean ± SD)	25.7 ± 5.1	26.5 ± 6.1
FM duration (years, mean ± SD)	9.6 ± 7.1	7.8 ± 6.2
Widespread Pain Index, ACR 2016 (0–19) (mean ± SD)	12.3 ± 3.1	12.8 ± 3.2
SSS, ACR 2016 (0–12) (mean ± SD)	8.6 ± 1.5	8.8 ± 1.8
DASS-42 stress score (0–42) (mean ± SD)	30.8 ± 6.3	30.4 ± 5.8
Pain (numerical scale) (0–10) (mean ± SD)	6.0 ± 1.8	6.4 ± 1.5
FIRST (0–6) (mean ± SD)	5.4 ± 0.8	5.6 ± 0.8
FIQ (0–100) (mean ± SD)	59.7 ± 10.7	62.3 ± 14.5
BPI Pain severity (0–10) (mean ± SD)	5.4 ± 1.2	5.7 ± 1.2
BPI Pain interference (0–10) (mean ± SD)	5.8 ± 1.7	5.9 ± 2.1
BPI Pain Experience (0–10) (mean ± SD)	5.6 ± 1.2	5.8 ± 1.3
PSQI Global score (0–21) (mean ± SD)	11.5 ± 3.2	13.8 ± 3.2
PSQI Subjective sleep quality (0–3) (mean ± SD)	2.1 ± 0.7	2.2 ± 0.7
PSQI Sleep latency (0–3) (mean ± SD)	2.1 ± 0.9	2.4 ± 0.8
PSQI Sleep duration (0–3) (mean ± SD)	1.2 ± 0.9	1.8 ± 0.9
PSQI Sleep efficiency (0–3) (mean ± SD)	1.3 ± 1.2	2.0 ± 1.1
PSQI Sleep disturbance (0–3) (mean ± SD)	2.2 ± 0.6	2.1 ± 0.5
PSQI Use of sleep medication (0–3) (mean ± SD)	1.2 ± 1.4	1.5 ± 1.5
PSQI Daytime dysfunction (0–3) (mean ± SD)	1.5 ± 0.8	1.9 ± 0.8
SF12-Mental score (0–100) (mean ± SD)	31.6 ± 6.2	33.0 ± 9.3
SF12-Physical score (0–100) (mean ± SD)	30.9 ± 5.8	30.7 ± 5.7
FSS (9–63) (mean ± SD)	51.9 ± 10.3	50.0 ± 12.0
PCS Total (0–52) (mean ± SD)	29.7 ± 10.5	28.9 ± 13.1
PCS Rumination (0–16) (mean ± SD)	9.3 ± 4.1	8.6 ± 4.9
PCS Magnification (0–12) (mean ± SD)	5.3 ± 2.8	6.1 ± 3.5
PCS Helplessness (0–24) (mean ± SD)	15.2 ± 4.9	14.2 ± 6.1
Precariousness (EPICES), (10–75) (mean ± SD)	26.9 ± 21.0	24.0 ± 19.8
Serum Mg (mmol/L) (mean ± SD)	0.92 ± 0.08	0.91 ± 0.08
Erythrocyte Mg (mmol/L) (mean ± SD)	2.86 ± 0.32	2.97 ± 0.31

**Table 2:** m/M and S/S+ subgroup parameters.

	m/M Subgroup (*n* = 16)	S/S+ Subgroup (*n* = 59)	*p*
Mean FM duration (mean ± SD)	6.1 ± 5.1	9.3 ± 7.0	0.09
Age < 50 years (percent)	50	34	0.5
Age ≥ 50 years (percent)	50	66	0.6
Pain (numerical scale) (0–10) (mean ± SD)	6.2 ± 1.6	6.2 ± 1.7	0.9
BPI Pain severity (0–10) (mean ± SD)	5.5 ± 1.0	5.6 ± 1.3	0.7

**Table 3:** Plasma and erythrocyte Mg concentrations after treatment (*n* = 75).

	Mg (*n* = 37)	Placebo (*n* = 38)	*p*
Serum Mg (mmol/L) (mean ± SD)	0.87 ± 0.07	0.87 ± 0.08	0.422
Erythrocyte Mg (mmol/L) (mean ± SD)	2.87 ± 0.37	2.91 ± 0.37	0.875

**Table 4:** Drug initiation after the beginning of the study (*n* = 75).

	Mg (*n* = 37)	Placebo (*n* = 38)	*p*
WHO level I analgesics (percent (number))	46.0 (17)	29.0 (11)	0.13
WHO level II analgesics (percent (number))	13.5 (5)	2.6 (1)	0.11
WHO level III analgesics (percent (number))	2.7 (1)	0 (0)	0.49
Co-analgesics (percent (number))	2.7 (1)	7.9 (3)	0.62
Antidepressants (percent (number))	10.8 (4)	7.9 (3)	0.71
Antiepileptics (percent (number))	0 (0)	2.6 (1)	1.00
Hypnotics (percent (number))	5.4 (2)	0 (0)	0.24
Anxiolytics (percent (number))	2.7 (1)	7.9 (3)	0.62

## Data Availability

The authors confirm that the data supporting the findings of this study are available within the article and its Supplementary Material.

## Supplementary Materials

The following supporting information can be downloaded at: https://www.mdpi.com/article/10.3390/nu14102088/s1, Table S1: secondary outcomes.

## References

[1] Heidari F., Afshari M., Moosazadeh M. (2017). Prevalence of Fibromyalgia in General Population and Patients, a Systematic Review and Meta-Analysis. Rheumatol. Int..

[2] Theoharides T.C., Tsilioni I., Arbetman L., Panagiotidou S., Stewart J.M., Gleason R.M., Russell I.J. (2015). Fibromyalgia Syndrome in Need of Effective Treatments. J. Pharmacol. Exp. Ther..

[3] Chinn S., Caldwell W., Gritsenko K. (2016). Fibromyalgia Pathogenesis and Treatment Options Update. Curr. Pain Headache Rep..

[4] Doerr J.M., Fischer S., Nater U.M., Strahler J. (2017). Influence of Stress Systems and Physical Activity on Different Dimensions of Fatigue in Female Fibromyalgia Patients. J. Psychosom. Res..

[5] Jiao J., Cheng Z., Wang W., Zhao Y., Jiang Q. (2021). Demographic Characteristics and Clinical Features of Fibromyalgia in China: A Cross-Sectional Study. Rheumatol. Ther..

[6] Martinez-Lavin M. (2007). Biology and Therapy of Fibromyalgia. Stress, the Stress Response System, and Fibromyalgia. Arthritis Res. Ther..

[7] McEwen B.S., Akil H. (2020). Revisiting the Stress Concept: Implications for Affective Disorders. J. Neurosci..

[8] Pickering G., Mazur A., Trousselard M., Bienkowski P., Yaltsewa N., Amessou M., Noah L., Pouteau E. (2020). Magnesium Status and Stress: The Vicious Circle Concept Revisited. Nutrients.

[9] Noushad S., Ahmed S., Ansari B., Mustafa U.-H., Saleem Y., Hazrat H. (2021). Physiological Biomarkers of Chronic Stress: A Systematic Review. Int. J. Health Sci..

[10] Gupta A., Silman A.J. (2004). Psychological Stress and Fibromyalgia: A Review of the Evidence Suggesting a Neuroendocrine Link. Arthritis Res. Ther..

[11] Adler G.K., Geenen R. (2005). Hypothalamic-Pituitary-Adrenal and Autonomic Nervous System Functioning in Fibromyalgia. Rheum. Dis. Clin. N. Am..

[12] Alok R., Das S.K., Agarwal G.G., Salwahan L., Srivastava R. (2011). Relationship of Severity of Depression, Anxiety and Stress with Severity of Fibromyalgia. Clin. Exp. Rheumatol..

[13] Malin K., Littlejohn G.O. (2013). Stress Modulates Key Psychological Processes and Characteristic Symptoms in Females with Fibromyalgia. Clin. Exp. Rheumatol..

[14] Taylor A.G., Fischer-White T.G., Anderson J.G., Adelstein K.E., Murugesan M., Lewis J.E., Scott M.M., Gaykema R.P.A., Goehler L.E. (2016). Stress, Inflammation and Pain: A Potential Role for Monocytes in Fibromyalgia-Related Symptom Severity. Stress Health J. Int. Soc. Investig. Stress.

[15] Rico-Villademoros F., Postigo-Martin P., Garcia-Leiva J.M., Ordoñez-Carrasco J.L., Calandre E.P. (2020). Patterns of Pharmacologic and Non-Pharmacologic Treatment, Treatment Satisfaction and Perceived Tolerability in Patients with Fibromyalgia: A Patients’ Survey. Clin. Exp. Rheumatol..

[16] Thour A., Marwaha R. (2021). Amitriptyline. StatPearls.

[17] Seelig M.S. (1994). Consequences of Magnesium Deficiency on the Enhancement of Stress Reactions; Preventive and Therapeutic Implications (a Review). J. Am. Coll. Nutr..

[18] Cuciureanu M.D., Vink R., Vink R., Nechifor M. (2011). Magnesium and Stress. Magnesium in the Central Nervous System.

[19] Costello R., Wallace T.C., Rosanoff A. (2016). Magnesium. Adv. Nutr..

[20] Pickering G., Morel V., Simen E., Cardot J.-M., Moustafa F., Delage N., Picard P., Eschalier S., Boulliau S., Dubray C. (2011). Oral Magnesium Treatment in Patients with Neuropathic Pain: A Randomized Clinical Trial. Magnes. Res..

[21] Bradley L.A. (2009). Pathophysiology of Fibromyalgia. Am. J. Med..

[22] Abraham G.E., Flechas J.D. (1992). Management of Fibromyalgia: Rationale for the Use of Magnesium and Malic Acid. J. Nutr. Med..

[23] Bagis S., Karabiber M., As I., Tamer L., Erdogan C., Atalay A. (2013). Is Magnesium Citrate Treatment Effective on Pain, Clinical Parameters and Functional Status in Patients with Fibromyalgia?. Rheumatol. Int..

[24] Engen D.J., McAllister S.J., Whipple M.O., Cha S.S., Dion L.J., Vincent A., Bauer B.A., Wahner-Roedler D.L. (2015). Effects of Transdermal Magnesium Chloride on Quality of Life for Patients with Fibromyalgia: A Feasibility Study. J. Integr. Med..

[25] Kim Y.-S., Kim K.-M., Lee D.-J., Kim B.-T., Park S.-B., Cho D.-Y., Suh C.-H., Kim H.-A., Park R.-W., Joo N.-S. (2011). Women with Fibromyalgia Have Lower Levels of Calcium, Magnesium, Iron and Manganese in Hair Mineral Analysis. J. Korean Med. Sci..

[26] Kasim A.A. (2011). Calcium, Magnesium and Phosphorous Levels in Serum of Iraqi Women with Fibromyalgia. Iraqi J. Pharm. Sci..

[27] Boulis M., Boulis M., Clauw D. (2021). Magnesium and Fibromyalgia: A Literature Review. J. Prim. Care Community Health.

[28] Andretta A., Dias Batista E., Madalozzo Schieferdecker M.E., Rasmussen Petterle R., Boguszewski C.L., Dos Santos Paiva E. (2019). Relation between Magnesium and Calcium and Parameters of Pain, Quality of Life and Depression in Women with Fibromyalgia. Adv. Rheumatol. Lond. Engl..

[29] Russell I.J., Michalek J.E., Flechas J.D., Abraham G.E. (1995). Treatment of Fibromyalgia Syndrome with Super Malic: A Randomized, Double Blind, Placebo Controlled, Crossover Pilot Study. J. Rheumatol..

[30] Ng S.Y. (1999). Hair Calcium and Magnesium Levels in Patients with Fibromyalgia: A Case Center Study. J. Manip. Physiol. Ther..

[31] Porter N.S., Jason L.A., Boulton A., Bothne N., Coleman B. (2010). Alternative Medical Interventions Used in the Treatment and Management of Myalgic Encephalomyelitis/Chronic Fatigue Syndrome and Fibromyalgia. J. Altern. Complement. Med..

[32] Shin H.-J., Na H.-S., Do S.-H. (2020). Magnesium and Pain. Nutrients.

[33] Tarasov E.A., Blinov D.V., Zimovina U.V., Sandakova E.A. (2015). Magnesium deficiency and stress: Issues of their relationship, diagnostic tests, and approaches to therapy. Ter. Arkh..

[34] Mohabbat A.B., Mahapatra S., Jenkins S.M., Bauer B.A., Vincent A., Wahner-Roedler D.L. (2019). Use of Complementary and Integrative Therapies by Fibromyalgia Patients: A 14-Year Follow-Up Study. Mayo Clin. Proc. Innov. Qual. Outcomes.

[35] Wolfe F., Clauw D.J., Fitzcharles M.-A., Goldenberg D.L., Häuser W., Katz R.L., Mease P.J., Russell A.S., Russell I.J., Walitt B. (2016). 2016 Revisions to the 2010/2011 Fibromyalgia Diagnostic Criteria. Semin. Arthritis Rheum..

[36] Bennett R. (2005). The Fibromyalgia Impact Questionnaire (FIQ): A Review of Its Development, Current Version, Operating Characteristics and Uses. Clin. Exp. Rheumatol..

[37] Perrot S., Bouhassira D., Fermanian J. (2010). Development and Validation of the Fibromyalgia Rapid Screening Tool (FiRST). Pain.

[38] Lovibond P.F., Lovibond S.H. (1995). The Structure of Negative Emotional States: Comparison of the Depression Anxiety Stress Scales (DASS) with the Beck Depression and Anxiety Inventories. Behav. Res. Ther..

[39] Cleeland C.S., Ryan K.M. (1994). Pain Assessment: Global Use of the Brief Pain Inventory. Ann. Acad. Med..

[40] Buysse D.J., Reynolds C.F., Monk T.H., Berman S.R., Kupfer D.J. (1989). The Pittsburgh Sleep Quality Index: A New Instrument for Psychiatric Practice and Research. Psychiatry Res..

[41] Ware J.E., Kosinski M., Keller S.D. (1996). A 12-Item Short-Form Health Survey: Construction of Scales and Preliminary Tests of Reliability and Validity. Med. Care.

[42] Ware J.E., Sherbourne C.D. (1992). The MOS 36-Item Short-Form Health Survey (SF-36). I. Conceptual Framework and Item Selection. Med. Care.

[43] Krupp L.B., LaRocca N.G., Muir-Nash J., Steinberg A.D. (1989). The Fatigue Severity Scale. Application to Patients with Multiple Sclerosis and Systemic Lupus Erythematosus. Arch. Neurol..

[44] Sullivan M.J.L., Bishop S.R., Pivik J. (1995). The Pain Catastrophizing Scale: Development and Validation. Psychol. Assess..

[45] Sass C., Dupré C., Giordanella J.P., Girard F., Guenot C., Labbe É., Rosa E.L., Magnier P., Martin É., Royer B. (2006). Le score Epices: Un score individuel de précarité. Construction du score et mesure des relations avec des données de santé, dans une population de 197 389 personnes. Bull. Epidemiol. Hebd..

[46] Committee for Proprietary Medicinal Products (CPMP) (2004). Points to Consider on Adjustment for Baseline Covariates. Stat. Med..

[47] Ruwaard J., Lange A., Bouwman M., Broeksteeg J., Schrieken B. (2007). E-Mailed Standardized Cognitive Behavioural Treatment of Work-Related Stress: A Randomized Controlled Trial. Cogn. Behav. Ther..

[48] Dualé C., Cardot J.-M., Joanny F., Trzeciakiewicz A., Martin E., Pickering G., Dubray C. (2018). An Advanced Formulation of a Magnesium Dietary Supplement Adapted for a Long-Term Use Supplementation Improves Magnesium Bioavailability: In Vitro and Clinical Comparative Studies. Biol. Trace Elem. Res..

[49] Eisinger J., Plantamura A., Marie P.A., Ayavou T. (1994). Selenium and Magnesium Status in Fibromyalgia. Magnes. Res..

[50] Romano T., Stiller J. (1994). Magnesium Deficiency in Fibromyalgia Syndrome. J. Nutr. Environ. Med..

[51] Costello R.B., Elin R.J., Rosanoff A., Wallace T.C., Guerrero-Romero F., Hruby A., Lutsey P.L., Nielsen F.H., Rodriguez-Moran M., Song Y. (2016). Perspective: The Case for an Evidence-Based Reference Interval for Serum Magnesium: The Time Has Come. Adv. Nutr..

[52] Batista E.D., Andretta A., de Miranda R.C., Nehring J., Paiva E.D.S., Schieferdecker M.E.M. (2016). Avaliação da ingestão alimentar e qualidade de vida de mulheres com fibromialgia. Rev. Bras. Reumatol..

[53] Elin R.J. (2010). Assessment of Magnesium Status for Diagnosis and Therapy. Magnes. Res..

[54] Barbagallo M., Veronese N., Dominguez L.J. (2021). Magnesium in Aging, Health and Diseases. Nutrients.

[55] Noah L., Pickering G., Mazur A., Dubray C., Hitier S., Dualé C., Pouteau E. (2020). Impact of Magnesium Supplementation, in Combination with Vitamin B6, on Stress and Magnesium Status: Secondary Data from a Randomized Controlled Trial. Magnes. Res..

[56] EFSA Panel on Dietetic Products, Nutrition and Allergies (NDA) (2015). Scientific Opinion on Dietary Reference Values for Magnesium. EFSA J..

[57] Morris M.E. (1992). Brain and CSF Magnesium Concentrations during Magnesium Deficit in Animals and Humans: Neurological Symptoms. Magnes. Res..

[58] Ross A.C., Caballero B.H., Cousins R.J., Tucker K.L., Ziegler T.R. (2012). Modern Nutrition in Health and Disease: Eleventh Edition.

[59] Botturi A., Ciappolino V., Delvecchio G., Boscutti A., Viscardi B., Brambilla P. (2020). The Role and the Effect of Magnesium in Mental Disorders: A Systematic Review. Nutrients.

[60] Benedetti F., Mayberg H.S., Wager T.D., Stohler C.S., Zubieta J.-K. (2005). Neurobiological Mechanisms of the Placebo Effect. J. Neurosci..

[61] Migliorini F., Maffulli N., Eschweiler J., Betsch M., Tingart M., Colarossi G. (2021). Placebo Effect in Pharmacological Management of Fibromyalgia: A Meta-Analysis. Br. Med. Bull..

[62] Kosek E., Rosen A., Carville S., Choy E., Gracely R.H., Marcus H., Petzke F., Ingvar M., Jensen K.B. (2017). Lower Placebo Responses After Long-Term Exposure to Fibromyalgia Pain. J. Pain.

[63] Chen X., Zou K., Abdullah N., Whiteside N., Sarmanova A., Doherty M., Zhang W. (2017). The Placebo Effect and Its Determinants in Fibromyalgia: Meta-Analysis of Randomised Controlled Trials. Clin. Rheumatol..

[64] Tseng M.-T., Chiang M.-C., Chao C.-C., Tseng W.-Y.I., Hsieh S.-T. (2013). FMRI Evidence of Degeneration-Induced Neuropathic Pain in Diabetes: Enhanced Limbic and Striatal Activations. Hum. Brain Mapp..

[65] Keulers E.H.H., Stiers P., Nicolson N.A., Jolles J. (2015). The Association between Cortisol and the BOLD Response in Male Adolescents Undergoing FMRI. Brain Res..

[66] Reicherts P., Wiemer J., Gerdes A.B.M., Schulz S.M., Pauli P., Wieser M.J. (2017). Anxious Anticipation and Pain: The Influence of Instructed vs Conditioned Threat on Pain. Soc. Cogn. Affect. Neurosci..

[67] Berretz G., Packheiser J., Kumsta R., Wolf O.T., Ocklenburg S. (2021). The Brain under Stress—A Systematic Review and Activation Likelihood Estimation Meta-Analysis of Changes in BOLD Signal Associated with Acute Stress Exposure. Neurosci. Biobehav. Rev..

[68] Peyron R., Faillenot I. (2011). Functional brain mapping of pain perception. Med. Sci..

[69] Garland E.L. (2012). Pain Processing in the Human Nervous System: A Selective Review of Nociceptive and Biobehavioral Pathways. Prim. Care.

[70] Garcia-Larrea L., Peyron R. (2013). Pain Matrices and Neuropathic Pain Matrices: A Review. Pain.

[71] Peyron R. (2014). Functional imaging of pain. Biol. Aujourdhui.

[72] Wager T.D., Rilling J.K., Smith E.E., Sokolik A., Casey K.L., Davidson R.J., Kosslyn S.M., Rose R.M., Cohen J.D. (2004). Placebo-Induced Changes in FMRI in the Anticipation and Experience of Pain. Science.

[73] Wager T.D., Atlas L.Y. (2015). The Neuroscience of Placebo Effects: Connecting Context, Learning and Health. Nat. Rev. Neurosci..

[74] Zunhammer M., Spisák T., Wager T.D., Bingel U. (2021). Meta-Analysis of Neural Systems Underlying Placebo Analgesia from Individual Participant FMRI Data. Nat. Commun..

[75] Vachon-Presseau E., Martel M.-O., Roy M., Caron E., Albouy G., Marin M.-F., Plante I., Sullivan M.J., Lupien S.J., Rainville P. (2013). Acute Stress Contributes to Individual Differences in Pain and Pain-Related Brain Activity in Healthy and Chronic Pain Patients. J. Neurosci..

[76] Van Houdenhove B., Egle U., Luyten P. (2005). The Role of Life Stress in Fibromyalgia. Curr. Rheumatol. Rep..

[77] Samartin-Veiga N., González-Villar A.J., Pidal-Miranda M., Vázquez-Millán A., Carrillo-de-la-Peña M.T. (2022). Active and Sham Transcranial Direct Current Stimulation (TDCS) Improved Quality of Life in Female Patients with Fibromyalgia. Qual. Life Res..

[78] Park R., Ho A.M.-H., Pickering G., Arendt-Nielsen L., Mohiuddin M., Gilron I. (2020). Efficacy and Safety of Magnesium for the Management of Chronic Pain in Adults: A Systematic Review. Anesth. Analg..

[79] Morel V., Pickering M.-E., Goubayon J., Djobo M., Macian N., Pickering G. (2021). Magnesium for Pain Treatment in 2021? State of the Art. Nutrients.

[80] Ali A., Njike V.Y., Northrup V., Sabina A.B., Williams A.-L., Liberti L.S., Perlman A.I., Adelson H., Katz D.L. (2009). Intravenous Micronutrient Therapy (Myers’ Cocktail) for Fibromyalgia: A Placebo-Controlled Pilot Study. J. Altern. Complement. Med..

[81] Ferreira I., Ortigoza Á., Moore P. (2019). Magnesium and malic acid supplement for fibromyalgia. Medwave.

[82] Calandre E.P., Morillas-Arques P., Molina-Barea R., Rodriguez-Lopez C.M., Rico-Villademoros F. (2011). Trazodone plus Pregabalin Combination in the Treatment of Fibromyalgia: A Two-Phase, 24-Week, Open-Label Uncontrolled Study. BMC Musculoskelet. Disord..

[83] Abdel Fattah Y.H., Elnemr R. (2020). Efficacy of Pregabalin as a Monotherapy versus Combined Pregabalin and Milnacipran in the Management of Fibromyalgia. Int. J. Rheum. Dis..

[84] Karamanlioglu D.S., Geler Kulcu D., Ozturk G., Akpinar P., Unlu Ozkan F., Aktas I. (2021). Effectiveness of Pregabalin Treatment for Trigger Points in Patients with Comorbid Myofascial Pain Syndrome and Fibromyalgia Syndrome: A Randomized Controlled Trial. Somatosens. Mot. Res..

[85] Lunn M.P., Hughes R.A., Wiffen P.J. (2014). Duloxetine for Treating Painful Neuropathy, Chronic Pain or Fibromyalgia. Cochrane Database Syst. Rev..

[86] Ghavidel-Parsa B., Bidari A., Rahimi A., Gharibpoor F., Khosousi M.-J. (2021). No Effect of Approved Fibromyalgia Drugs on the Social Pain (Invalidation) Contrary to Physical Pain: An Open-Label Short-Term Randomized Clinical Trial. Clin. Rheumatol..

[87] Häuser W., Petzke F., Üçeyler N., Sommer C. (2011). Comparative Efficacy and Acceptability of Amitriptyline, Duloxetine and Milnacipran in Fibromyalgia Syndrome: A Systematic Review with Meta-Analysis. Rheumatology.

[88] Macfarlane G.J., Kronisch C., Atzeni F., Häuser W., Choy E.H., Amris K., Branco J., Dincer F., Leino-Arjas P., Longley K. (2017). EULAR Recommendations for Management of Fibromyalgia. Ann. Rheum. Dis..

[89] Martínez-Rodríguez A., Rubio-Arias J.Á., Ramos-Campo D.J., Reche-García C., Leyva-Vela B., Nadal-Nicolás Y. (2020). Psychological and Sleep Effects of Tryptophan and Magnesium-Enriched Mediterranean Diet in Women with Fibromyalgia. Int. J. Environ. Res. Public. Health.

[90] Arab A., Rafie N., Amani R., Shirani F. (2022). The Role of Magnesium in Sleep Health: A Systematic Review of Available Literature. Biol. Trace Elem. Res..

[91] Pickering G., Macian N., Delage N., Picard P., Cardot J.-M., Sickout-Arondo S., Giron F., Dualé C., Pereira B., Marcaillou F. (2018). Milnacipran Poorly Modulates Pain in Patients Suffering from Fibromyalgia: A Randomized Double-Blind Controlled Study. Drug Des. Devel. Ther..

[92] Macian N., Pereira B., Shinjo C., Dubray C., Pickering G. (2015). Fibromyalgia, Milnacipran and Experimental Pain Modulation: Study Protocol for a Double Blind Randomized Controlled Trial. Trials.

