# Supplementary material for: Short-Term Magnesium Therapy Alleviates Moderate Stress in Patients with Fibromyalgia: A Randomized Double-Blind Clinical Trial
Source: Nutrients. 2022 May 17;14(10):2088. doi: 10.3390/nu14102088 (PMC9145501; doi:10.3390/nu14102088)
Supplement: Supplementary file 1 [file nutrients-14-02088-s001.zip › nutrients-1713333-supplementary.pdf]

Supplemental data table S1 – secondary outcomes

Medical history of patients (part I)

		Mg percent	Placebo percent
Cardiovascular	hypertension	19	13
	cardiac arrhythmias	8	5
	raynaud's disease	0	5
	surgery (varicose, saphenectomy)	8	11
	dyslipidemia	8	9
Respiratory	asthma	11	8
	chronic bronchitis	6	0
	histiocytosis	3	3
	pulmonary embolism	3	0
	COPD	8	0
	pneumonia	3	0
Gastrointestinal	bloating	0	3
	constipation	11	3
	gastroesophageal reflux	14	6
	hemorrhoids crisis	0	5
	hiatus hernia	8	5
	irritable bowel syndrome	5	21
	sigmoiditis	3	0
	surgery (appendectomy, polyps, cholecystectomy, inguinal hernia)	47	77
Hepatic	hepatic angioma	3	0
	hepatitis	3	3
	hepatic cytolysis	3	0
Urology	bladder surgery	3	0
	cystitis	0	5
	hyperuricemia	3	0
	pyelonephritis	3	8
	renal colic	0	3
	urinary incontinence	3	0
	vesical renal reflux	3	0

Supplemental data table S1 – secondary outcomes

Medical history of patients (part II)

		Mg percent	Placebo percent
Neurologic Psychiatric	anorexia	0	3
	anxiety	8	3
	balance problems	0	3
	bipolar disorders	5	0
	cruralgia	3	0
	depressive syndrom	16	32
	encephalitis	3	0
	headache	3	0
	insomnia	3	0
	ischemic stroke	0	3
	migraine	8	13
	mysathenia	3	0
	neuropathic pain	9	3
	sciatica	0	6
Genital	breast cancer surgery	6	0
	endometriosis	14	3
	papillomavirus infection	0	3
	salpingitis	5	3
	surgery(breast and ovorian cyst, hysterectomy, ovariectomy	30	25
Rheumatologic	algodystrophy	6	3
	arthrosis	5	13
	disc herniation	14	8
	Inflammatory diseases (polyarthritis, acute rheumatism crisis, spondylitis)	6	3
	osteoporosis	3	3
	scheuermann disease	0	3
	scoliosis	3	0
	surgery (arm, wrist, carpal tunnel)	16	8
Endocrinology Hematology	hodglin's lymphoma	3	0
	hyperthyroidism	0	3
	hypocalcemia	3	0
	hypothyroidism	32	24
	sepsis	3	0
	thyroid nodules	5	3

Supplemental data table S1 – secondary outcomes

Concomitants medications of patients at inclusion

**Class of treatment**	**Mg percent**	**Placebo percent**
High blood pressure	16.2	10.5
Statins	5.4	5.3
Calcium chanel blokers	10.8	2.6
Steroids	29.7	31.6
Laxatives	13.5	10.5
Proton pomp inhibitors	18.9	23.7
WHO level I analgesics	40.5	55.3
WHO level II analgesics	59.5	42.1
WHO level III analgesics	5.4	5.3
Co-analgesics	13.5	21.1
Antidepressants	54.1	42.1
Antiepileptics	8.1	13.2
Hypnotics	8.1	23.7
Anxiolytics	37.8	29.0

Supplemental data table S1 – secondary outcomes

Pain parameters and fibromyalgia specific questionnaires

	All patients			Patients with mild/moderate (m/M) score at inclusion			Patients with severe/extremely severe (S/S+) score at inclusion		
Parameters	Mg	Placebo	p	Mg	Placebo	p	Mg	Placebo	p
**Pain (numerical scale) (0-10)**									
INCLUSION	6.0±1.8	6.4±1.5	/	6.1±1.9	6.4±1.1	/	6.0±1.8	6.4±1.5	/
D0	5.6±1.8	5.9±1.9	0.892	6.4±1.9	5.9±1.3	0.256	5.3±1.6	5.9±2.0	0.672
D28	4.5±1.6	4.8±2.3	0.949	4.6±1.7	5.4±1.7	0.392	4.4±1.6	4.7±2.4	0.693
D84	5.2±2.0	5.6±2.0	0.908	5.4±1.5	6.3±1.3	0.417	5.2±2.2	5.4±2.2	0.693
**Fibromyalgia Impact Questionnaire (FIQ) (0-100)**									
D0	59.7±10.7	62.3±14.5	/	59.2±7.5	62.7±15.1	/	59.9±11.7	62.2±14.6	/
D28	55.7±12.5	53.1±19.7	0.048	52.4±12.8	56.4±15.6	0.936	56.8±12.4	52.3±20.7	0.026
D84	57.4±12.8	57.5±16.2	0.345	57.7±11.2	62.3±15.7	0.824	57.3±13.5	56.4±16.3	0.288
**Brief Pain Inventory (BPI) (0-10)** *Pain severity*									
D0	5.4±1.2	5.7±1.2	/	5.7±1.3	5.3±0.7	/	5.3±1.2	5.8±1.3	/
D28	5.0±1.6	5.1±1.8	0.545	5.1±1.7	5.6±1.2	0.029	4.9±1.6	5.1±1.9	0.169
D84	5.4±1.5	5.6±1.7	0.746	5.8±1.0	6.2±1.3	0.066	5.2±1.5	5.5±1.8	0.396
**Brief Pain Inventory (BPI) (0-10)** *Pain interference*									
D0	5.8±1.7	5.9±2.1	/	5.2±2.0	5.3±2.0	/	6.0±1.6	6.0±2.1	/
D28	5.4±1.8	5.1±2.2	0.266	5.1±1.4	5.8±1.6	0.297	5.4±1.9	5.0±2.3	0.152
D84	5.6±1.7	5.3±2.0	0.289	5.7±1.5	5.8±1.7	0.942	5.5±1.7	5.2±2.1	0.357
**Brief Pain Inventory (BPI) (0-10)** *Pain Experience*									
D0	5.6±1.2	5.8±1.3	/	5.3±1.3	5.3±1.0	/	5.7±1.1	5.9±1.4	/
D28	5.2±1.5	5.1±1.8	0.323	5.1±1.4	5.7±1.2	0.068	5.2±1.6	5.0±1.9	0.114
D84	5.5±1.3	5.5±1.7	0.450	5.8±0.9	6.0±1.3	0.361	5.4±1.4	5.4±1.7	0.354

Supplemental data table S1 – secondary outcomes

Sleep quality questionnaire (part I)

	All patients			Patients with mild/moderate (m/M) score at inclusion			Patients with severe/extremely severe (S/S+) score at inclusion		
Parameters	Mg	Placebo	p	Mg	Placebo	p	Mg	Placebo	p
**Pittsburgh Sleep Quality Index (PSQI) (0-21)** *Global score*									
D0	11.5±3.2	13.8±3.2	/	10.8±3.1	14.3±2.9	/	11.7±3.3	13.7±3.3	/
D28	11.4±3.5	12.5±3.3	0.052	9.8±1.9	13.3±2.3	1	11.9±3.7	12.4±3.5	0.026
D84	11.9±3.2	12.7±3.9	0.023	11.8±2.9	12.6±3.6	0.014	11.9±3.4	12.7±4.0	0.158
**Pittsburgh Sleep Quality Index (PSQI) (0-3)** *Subjective sleep quality*									
D0	2.1±0.7	2.2±0.7	/	2.3±0.7	2.0±0.0	/	2.0±0.7	2.3±0.7	/
D28	2.1±0.6	2.1±0.7	0.552	2.2±0.4	2.0±0.0	0.696	2.0±0.7	2.1±0.8	0.407
D84	2.2±0.7	2.2±0.7	0.522	2.4±0.5	2.0±0.8	0.696	2.1±0.8	2.3±0.7	0.613
**Pittsburgh Sleep Quality Index (PSQI) (0-3)** *Sleep latency*									
D0	2.1±0.9	2.4±0.8	/	1.8±1.0	2.0±0.8	/	2.2±0.9	2.5±0.8	/
D28	1.9±1.0	2.0±1.0	0.324	1.2±1.0	1.4±0.5	0.970	2.1±0.9	2.1±1.0	0.217
D84	2.1±0.9	2.2±1.0	0.375	1.6±1.0	1.9±0.9	0.852	2.1±0.8	2.2±1.0	0.254
**Pittsburgh Sleep Quality Index (PSQI) (0-3)** *Sleep duration*									
D0	1.2±0.9	1.8±0.9	/	1.3±1.3	1.9±1.1	/	1.1±0.8	1.7±0.9	/
D28	1.2±0.8	1.6±0.9	0.169	1.2±1.0	1.9±1.2	0.711	1.2±0.8	1.5±0.8	0.092
D84	1.2±0.9	1.7±1.0	0.621	1.1±1.1	1.7±1.4	0.792	1.2±0.9	1.6±1.0	0.463
**Pittsburgh Sleep Quality Index (PSQI) (0-3)** *Sleep efficiency*									
D0	1.3±1.2	2.0±1.1	/	1.2±1.3	2.0±1.4	/	1.3±1.2	2.0±1.1	/
D28	1.1±1.2	2.0±1.1	0.595	0.6±1.0	2.1±1.2	0.117	1.3±1.2	2.0±1.1	0.788
D84	1.2±1.3	1.9±1.3	0.956	1.2±1.5	1.6±1.5	0.406	1.1±1.2	1.9±1.3	0.737

Sleep quality questionnaire (part II)

Supplemental data table S1 – secondary outcomes

	All patients			Patients with mild/moderate (m/M) score at inclusion			Patients with severe/extremely severe (S/S+) score at inclusion		
Parameters	Mg	Placebo	p	Mg	Placebo	p	Mg	Placebo	p
**Pittsburgh Sleep Quality Index (PSQI) (0-3)**<br>*Sleep disturbance*									
D0	2.2±0.6	2.1±0.5	/	2.0±0.5	2.1±0.4	/	2.2±0.6	2.0±0.5	/
D28	2.1±0.5	2.0±0.6	0.708	2.1±0.6	2.0±0.6	0.222	2.1±0.5	2.0±0.6	0.892
D84	2.3±0.5	2.0±0.5	0.428	2.3±0.5	2.1±0.4	0.109	2.2±0.5	2.0±0.5	0.942
**Pittsburgh Sleep Quality Index (PSQI) (0-3)**<br>*Use of sleep medication*									
D0	1.2±1.4	1.5±1.5	/	1.0±1.5	2.1±1.5	/	1.2±1.4	1.3±1.4	/
D28	1.3±1.5	1.2±1.4	0.079	1.0±1.5	1.9±1.3	0.384	1.4±1.4	1.1±1.4	0.0.112
D84	1.4±1.3	1.1±1.4	0.005	1.1±1.5	1.6±1.5	0.038	1.5±1.3	1.0±1.4	0.022
**Pittsburgh Sleep Quality Index (PSQI) (0-3)**<br>*Daytime dysfunction*									
D0	1.5±0.8	1.9±0.8	/	1.1±0.8	2.1±0.9	/	1.7±0.8	1.9±0.8	/
D28	1.7±0.7	1.7±0.9	0.019	1.4±0.5	2.0±0.8	0.191	1.8±0.7	1.6±0.9	0.041
D84	1.7±0.7	1.7±0.9	0.085	2.0±0.7	1.7±0.8	0.000	1.6±0.6	1.7±0.9	0.978

Supplemental data table S1 – secondary outcomes

Quality of life and fatigue questionnaire

	All patients			Patients with mild/moderate (m/M) score at inclusion			Patients with severe/extremely severe (S/S+) score at inclusion		
Parameters	Mg	Placebo	p	Mg	Placebo	p	Mg	Placebo	p
**Quality of life – short form 12 (SF-12) (0-100)** *Mental score*									
D0	31.6±6.2	33.0±9.3	/	32.5±5.3	35.4±13.9	/	31.3±6.5	32.5±8.1	/
D28	33.8±7.2	34.9±9.8	0.930	36.3±7.5	36.7±12.0	0.380	33.0±7.1	34.5±9.4	0.735
D84	33.2±7.0	35.7±9.3	0.483	34.8±7.1	37.3±12.2	0.883	32.7±7.1	35.3±8.7	0.403
**Quality of life – short form 12 (SF-12) (0-100)** *Physical score*									
D0	30.9±5.8	30.7±5.7	/	30.2±6.0	28.5±4.2	/	31.2±5.8	31.2±5.9	/
D28	31.8±5.7	31.1±6.8	0.589	29.5±5.1	27.9±3.9	0.980	32.5±5.8	31.8±7.2	0.459
D84	32.0±5.9	31.0±6.1	0.381	28.3±6.2	29.7±3.3	0.108	33.3±5.3	31.3±6.6	0.056
**Fatigue Severity Scale (FSS) (9-63)**									
D0	51.9±10.3	50.0±12.0	/	51.3±7.8	51.9±10.8	/	52.1±11.1	49.6±12.4	/
D28	49.9±12.2	47.2±13.1	0.348	49.7±6.3	50.7±11.2	0.816	50.0±13.6	46.3±13.5	0.305
D84	50.1±11.1	48.4±12.9	0.536	49.3±9.2	50.4±12.6	0.843	50.4±11.8	47.9±13.2	0.458

Supplemental data table S1 – secondary outcomes

Catastrophising and precariousness scales

	All patients			Patients with mild/moderate (m/M) score at inclusion			Patients with severe/extremely severe (S/S+) score at inclusion		
Parameters	Mg	Placebo	p	Mg	Placebo	p	Mg	Placebo	p
**Pain Catastrophing Scale (PCS) (0-52)** *Total*									
D0	29.7±10.5	28.9±13.1	/	30.3±7.3	27.1±11.6	/	29.5±11.4	29.3±13.5	/
D28	27.5±12.7	27.2±13.1	0.654	28.1±11.1	26.1±12.7	0.509	27.3±13.4	27.4±13.3	0.896
D84	27.1±13.2	26.1±14.2	0.877	29.1±10.7	26.9±10.7	0.557	26.4±14.0	26.0±15.0	0.924
**Pain Catastrophing Scale (PCS) (0-16)** *Rumination*									
D0	9.3±4.1	8.6±4.9	/	9.8±2.7	8.3±4.4	/	9.1±4.4	8.7±5.1	/
D28	9.1±4.4	8.2±4.5	0.849	10.1±3.3	8.4±4.3	0.859	8.7±4.7	8.1±4.6	0.794
D84	8.9±4.8	8.4±4.5	0.750	9.8±4.1	9.7±3.3	0.218	8.6±5.1	8.1±4.8	0.864
**Pain Catastrophing Scale (PCS) (0-12)** *Magnification*									
D0	5.3±2.8	6.1±3.5	/	4.9±2.5	6.1±2.9	/	5.4±2.9	6.1±3.7	/
D28	4.8±3.5	5.5±3.3	0.938	3.9±2.9	5.3±3.7	0.589	5.0±3.7	5.5±3.3	0.737
D84	4.7±3.5	4.9±3.4	0.384	4.6±3.1	5.3±2.8	0.987	4.8±3.6	4.9±3.5	0.301
**Pain Catastrophing Scale (PCS) (0-24)** *Helplessness*									
D0	15.2±4.9	14.2±6.1	/	15.6±3.5	12.7±4.6	/	15.0±5.3	14.5±6.5	/
D28	13.6±6.1	13.5±6.3	0.356	14.1±5.8	12.4±5.4	0.415	13.5±6.2	13.8±6.5	0.529
D84	13.5±6.1	12.8±7.1	0.617	14.8±5.3	11.9±5.3	0.873	13.0±6.4	13.0±7.5	0.603
**Precariousness (EPICES) (10-75)**									
D0	26.9±21.0	24.0±19.8	/	/	/	/	/	/	/
D28	24.7±19.7	23.2±20.5	0.079						
D84	26.1±20.1	23.5±20.3	0.636						

Supplemental data table S1 – secondary outcomes

Mg assays

	All patients			Patients with mild/moderate (m/M) score at inclusion			Patients with severe/extremely severe (S/S+) score at inclusion		
Parameters	Mg	Placebo	p	Mg	Placebo	p	Mg	Placebo	p
**Serum Mg (mmol/l)**									
INCLUSION	0.92±0.08	0.91±0.08	/	0.96±0.08	0.90±0.07	/	0.91±0.07	0.91±0.08	/
D28	0.87±0.07	0.87±0.08	0.422	0.88±0.04	0.86±0.06	0.319	0.87±0.07	0.87±0.09	0.778
D84	0.87±0.08	0.88±0.08	0.134	0.88±0.08	0.86±0.04	0.369	0.87±0.08	0.89±0.08	0.292
**Erythrocyte Mg (mmol/l)**									
INCLUSION	2.86±0.32	2.97±0.31	/	3.10±0.25	2.90±0.33	/	2.79±0.31	2.98±0.31	/
D28	2.87±0.37	2.91±0.37	0.875	2.79±0.33	3.00±0.35	0.000	2.90±0.38	2.89±0.39	0.024
D84	2.94±0.38	2.91±0.36	0.212	3.05±0.29	2.97±0.32	0.313	2.90±0.41	2.89±0.37	0.031
